# New Method for Differentiation of Granuloviruses (Betabaculoviruses) Based on Real-Time Polymerase Chain Reaction (Real-Time PCR)

**DOI:** 10.3390/v11020115

**Published:** 2019-01-29

**Authors:** Martyna Krejmer-Rabalska, Lukasz Rabalski, Michael D. Jukes, Marlinda Lobo de Souza, Sean D. Moore, Boguslaw Szewczyk

**Affiliations:** 1 Laboratory of Recombinant Vaccines, Intercollegiate Faculty of Biotechnology of University of Gdansk and Medical University of Gdansk, University of Gdansk, 80-307 Gdansk, Poland; martyna.krejmer@biotech.ug.edu.pl (M.K.-R.); boguslaw.szewczyk@biotech.ug.edu.pl (B.S.); 2 Department of Biochemistry and Microbiology, Rhodes University, P.O. Box 94, Grahamstown 6140, South Africa; mrmdjukes@gmail.com; 3 Centre for Biological Control, Department of Zoology and Entomology, Rhodes University, P.O. Box 94, Grahamstown 6140, South Africa; seanmoore@cri.co.za; 4 Embrapa Recursos Genéticos e Biotecnologia, Parque Estacao Biológica, Brasilia 70770-900, Brazil; marlinda.souza@embrapa.br; 5 Citrus Research International (CRI), P.O. Box 5095, Walmer 6065, Port Elizabeth, South Africa

**Keywords:** betabaculovirus, detection, real-time PCR, *lef-9*, *lef-8*, granulin

## Abstract

*Baculoviridae* is a highly diverse family of rod-shaped viruses with double-stranded DNA. To date, almost 100 species have had their complete genomic sequences deposited in the GenBank database, a quarter of which comprises granuloviruses (GVs). Many of the genomes are sequenced using next-generation sequencing, which is currently considered the best method for characterizing new species, but it is time-consuming and expensive. Baculoviruses form a safe alternative to overused chemical pesticides and therefore there is a constant need for identifying new species that can be active components of novel biological insecticides. In this study, we have described a fast and reliable method for the detection of new and differentiation of previously analyzed granulovirus species based on a real-time polymerase chain reaction (PCR) technique with melting point curve analysis. The sequences of highly conserved baculovirus genes, such as *granulin* and *late expression factors 8* and *9* (*lef-8* and *lef-9*), derived from GVs available to date have been analyzed and used for degenerate primer design. The developed method was tested on a representative group of eight betabaculoviruses with comparisons of melting temperatures to allow for quick and preliminary granulovirus detection. The proposed real-time PCR procedure may be a very useful tool as an easily accessible screening method in a majority of laboratories.

## 1. Introduction

Insect pests pose a threat for crops and forests all over the world. To manage them, chemical control agents are usually applied, and are very often overused, because an immediate effect is sought. To reduce chemicals in the environment, there is a constant need for finding novel biologically safe alternatives. Some very promising candidates for biopesticides belong to the *Baculoviridae* family, which is currently the biggest insect virus family, comprising around 1000 described species of insect viruses [1,2,3]. Baculoviruses are known to naturally control many insect populations. They not only possess a very narrow host range but also do not accumulate in the environment and, being selective, are safe for humans and animals. They have been applied in the field with greater or lesser success since the 1940s, but in the last two decades, they have once again gained popularity [3,4].

The *Baculoviridae* family consists of viruses with double-stranded DNA, covalently closed, circular genomes [5] that range in size from 82 kilo-base pair (kbp) (*Neodiprion lecontei* nucleopolyhedrovirus (NeleNPV) [6]) to 179 kbp (*Xestia c-nigrum* granulovirus (XecnGV) [7]) and encode between 89 and 181 open reading frames (orfs). Based on common structural features, phylogeny, and host taxonomy, baculoviruses are divided into four genera. Of these, the genus *Betabaculovirus* contains all known lepidopteran-specific granuloviruses. Its representatives differ from members of the remaining three genera—*Alphabaculovirus, Deltabaculovirus*, and *Gammabaculovirus*—because of smaller, ovo-cylindrical occlusion bodies consisting of the major occlusion protein, granulin, which is a highly conserved structural protein [8,9,10]. The life cycle of granuloviruses (GVs) is similar to that of alphabaculoviruses, while little detail is known about the remaining two genera [11]. Betabaculoviruses are reported to have different tissue tropism and, on this basis, are divided into three groups [12]. To the first group belong the slow-killing GVs that infect the insect midgut epithelium and fat tissue. Representatives include XcGV [7] or *Helicoverpa armigera* granulovirus (HearGV) [13]. The second group consists of fast-killing GVs, including *Cydia pomonella* granulovirus (CpGV) [14], *Cryptophlebia leucotreta* granulovirus (CrleGV) [15,16] and *Epinotia aporema* granulovirus (EpapGV) [17]. In this group, the infection spreads in most tissues of the caterpillar in a similar manner to alphabaculovirus infections, resulting in the fast death of an infected larvae. The third group is known only for *Harrisina brillians* granulovirus (HabrGV) [18] and is restricted to the midgut epithelium. This group is highly infectious, causing diarrhea in infected larvae, resulting in the quick spread of infection in larval populations [19]. The complete genome of HabrGV remains unknown [20] (GenBank, 2018).

All members of the *Baculoviridae* family retain a set of 38 core genes conserved in all genomic sequences, with this set of common genes playing an important role in the baculovirus life cycle. These genes are involved in viral functions such as transcription, DNA replication, virion structure, and impairing cellular metabolism during infection [21,22,23]. For example, the *late expression factor 8* and *9 (lef-8 and lef-9)* core genes code for viral RNA polymerase subunits, which regulate transcription of late and very late viral genes [11,24].

Nearly 100 genomes of the *Baculoviridae* family species have been sequenced and deposited in GenBank to date, of which 24 are granuloviruses (GenBank, 2018). At present, the most reliable technique for detection and confirmation of new species is Sanger sequencing of fragments of *gran, lef-8* and *lef-9* genes [25]. This method is based on generating (during specific PCR conditions for every gene) amplicons and further sequencing. Its main drawback is an obligatory nucleotide sequence analysis which cannot be performed in a majority of laboratories and also generates extra costs. Another technique that can be used for new species detection is a whole genome sequencing (no need for specific primers/PCR). Unfortunately, Next Generation Sequencing (NGS) technology has some disadvantages. Although it becomes less expensive each consecutive year, the cost is still very high, and the procedure includes time-consuming sample preparation and data processing. Furthermore, it cannot be used routinely in screening samples from the field because there is a need for long and complicated library preparations [26].

It was reported that one insect host species may be infected with a few different baculoviruses [27,28] or, conversely, one virus can have different hosts [29], indicating that an efficient screening method for mixed populations of multiple baculoviruses may be useful. Methods based on PCR utilizing *granulin* or *polyhedrin* sequences with specific primers enables simple detection of known NPVs or GVs in a sample and has been commonly used in many laboratories. PCR amplification followed by restriction fragment length polymorphism (PCR-RFLP), with degenerate primers for the *polyhedrin* fragment, was also used for the identification of a few NPVs [30]. Subsequently, a PCR-based method for the detection and identification of different species was established for NPVs and GVs, but the sequencing of products was necessary [31]. All these methods rely on the identification of different species at the end-point and not during the reaction. Fast differentiation of a few (or completely new) species in the environmental sample may be quite complex. In the past, our team has developed a method for the differentiation of nucleopolyhedroviruses [32] and, more recently, for granuloviruses [33], both based on the Multi-temperature Single-Stranded Conformational Polymorphism (MSSCP) technique [34]. However, in the current study, we report a method based on real-time polymerase chain reaction (real-time PCR) that allows for differentiation between betabaculoviruses. The method can be complimentary to the MSSCP method or it can be used to screen large sample sets for a variety of granuloviruses without requiring sequencing of each amplicon generated.

## 2. Materials and Methods

### 2.1. Phylogenetic Analysis of Granuloviruses

The 38 core genes were extracted from nucleotide sequences of genomes of each betabaculovirus available in the GenBank database and were translated into amino acid sequences using Geneious Pro 7.1 (Biomatters, Auckland, New Zealand). Then, the MAFFT alignment was performed for each gene from all the known species. Subsequently, alignments were concatenated and the maximum-likelihood (ML) method applied with 1000 bootstrap replicates (percentage of replicates in which the associated *Baculoviridae* members clustered together is presented next to the branches) used to construct a phylogenetic tree using MEGA 7 software. GenBank accession numbers of available betabaculovirus species are presented in Table 1. The alphabaculovirus *Autographa californica* multiple nucleopolyhedrovirus (AcMNPV; L22858), deltabaculovirus *Culex nigripalpus* nucleopolyhedrovirus (CuniNPV; AF403738), and gammabaculovirus *Neodiprion sertifer* nucleopolyhedrovirus (NeseNPV; AY430810) were added to demonstrate the general relationship in the *Baculoviridae* family.

### 2.2. Determination of Betabaculovirus Representative Group

A representative group of eight betabaculoviruses were chosen for further studies on the basis of the phylogenetic tree (Section 2.1) and meeting the following criteria: (i) different *grannulin, lef-8* and *lef-9* sequences, (ii) different clades among the genera, (iii) distant from each other in the phylogenetic tree, (iv) a pair of closely related species included, and (v) DNA availability.

### 2.3. Virus Purification and DNA Isolation

Viruses were purified from crude larval homogenates through filtration and then centrifugation at 5000× *g* for 10 min at room temperature. The supernatant was discarded, and a 200 µL of ddH_2_O was added to the pellet and centrifuged again as before. The supernatant was discarded, and the pellet with granules was re-suspended in 0.5% SDS and centrifuged as before. In the next step, the pellet was re-suspended in 0.5 M NaCl and the centrifugation was repeated. Thereafter, the granules in the pellet were re-suspended in a 200 µL of ddH_2_O. Such samples were then dissolved in an alkaline solution (0.1 M Na2CO3, pH 10.0) for 30 min. The solution was then neutralized with an equal amount of Tris-HCl buffer (pH 6.4). The DNA extraction was performed with the use of the MagAttract HMW DNA Kit (Qiagen, Venlo, The Netherlands) according to the manufacturer’s protocol. The DNA concentration was determined using a Quantus Fluorometer (Promega, Madison, WI, USA) and its dedicated Qubit dsDNA HS (High Sensitivity) Assay Kit (Thermo Fisher Scientific, Waltham, MA, USA).

### 2.4. Granulin, Late Expression Factor-9 and Late Expression Factor-8 Nucleotide Sequence Alignments and Degenerate Primer Design; Amino Acid Alignment of the PCR Products.

The nucleotide sequence alignments based on whole genes, *granulin, late expression factor-9 (lef-9),* and *late expression factor-8 (lef-8)*, were extracted from the genome sequences of betabaculoviruses (24 species) available in the GenBank database (Table 1) using Geneious Pro 7.1 (Biomatters, Auckland, New Zealand) with default settings. Prior to primer design for real-time PCR, the alignments were manually analyzed to choose the most variable region occurring between the two most conserved sequences while also being around 100–250 bp in length. Although *gran, lef-9* and *lef-8* share high levels of similarity between GVs, there are some differences in the nucleotide sequences, and to therefore enable the detection of different species, it is necessary to design degenerate universal primers. The primers used for short fragments of *granulin* (125 bp) and *lef-9* (179 bp) previously published by Krejmer-Rabalska et al. [33] (gran-F–5′TAC ATG GTB ACN GAR GA3′, Tm = 43.4–52.2 °C, gran-R-5′AAY TCY TTN CCG CTC CAG TT3′, Tm = 51.9–59.4 °C; lef-9-F-5′CAR AAC AAR AAY GGR TAY GC3′, Tm = 45.9–56.1 °C, lef-9-R-5′GGR TGN CGH GTG TTC CAY AC3′, Tm = 52.7–64.3 °C) were re-evaluated against a broader group of betabaculovirus species (all 24 available, Figure 3a,b) for use in real-time PCR reactions. Degenerate primers were designed to amplify a short fragment of *lef-8* (119 bp)—lef-8-F-5′CCK TAY ATK TTY TTY AAC AA3′, Tm = 39–50.5 °C, lef-8-R-5′GAT TGA TTD ATR CTC CA3′, Tm = 39.2–46.4 °C (Figure 3c; IUPAC genetic code: B–C or G or T, N–A or T or C or G; R–A or G, Y–C or T, H–A or C or T, K–G or T, D–A or G or T).

The nucleotide sequences of the predicted PCR products were extracted from the various genomic sequences along with one or two additional nucleotides (to maintain the correct open reading frame), translated into amino acid sequences, and aligned using Geneious Pro 7.1 (Biomatters, Newark, NJ, USA), with forward and reverse primers shown (Figure S1).

### 2.5. Real-Time PCR Reaction

Real-time PCR was performed using a LightCycler 480 Instrument II (Roche, Germany). DNA templates from eight granuloviruses and one additional CrleGV isolate were subjected to real-time PCR with the use of SG qPCR Master Mix (2×) (EURX, Poland) for each of the three pairs of primers to amplify the *granulin*, *lef-9*, and *lef-8* short fragments. The annealing temperature was optimized to be the same for all three primer pairs (*gran*, *lef-9*, and *lef-8*).

The final mixture of 25 µL (prepared on a 96-well plate) contained 12.5 µL of Master Mix (2×) (Perpetual Taq DNA Polymerase, reaction buffer with 2.5 mM MgCl_2_, dNTPs and SYBR Green I dye), 0.3 μM of each primer, and 0.6 μl DNA template (0.5 ng in total). A negative control (with alphabaculovirus *Lymantria dispar* nucleopolyhedrovirus (LdMNPV) DNA) was prepared as well. The samples were initially stored at 50 °C for 2 min to activate the hot start polymerase, then denatured at 95 °C for 10 min, followed by 45 cycles: 94 °C (denaturation) for 15 s, 50 °C (annealing) for 30 s, and 72 °C (extension) for 30 s. To confirm the specificity of the amplified product, a melting curve analysis step was included. The internal temperature of the Light Cycler was increased rapidly to 95 °C, then decreased to 65 °C, and then the sample was incubated for 1 min. The fluorescence at 530 nm was measured continuously. The melting peaks were generated using Light Cycler software by plotting the first negative derivative of the fluorescence over the temperature versus the temperature (−d*F*/d*T*). To confirm the presence of specific products, agarose electrophoresis (2%) was performed. All the reactions were repeated three times with the mean Tm calculated from all the repetitions of the experiment, and standard deviation (SD) was also calculated.

Additionally, serial dilutions from 10^−1^–10^−10^ of HearGV DNA were prepared to check the detection level of the method. Following real-time PCR amplification, DNA concentrations for each reaction were measured using the fluorometric method (Qubit ssDNA HS Assay Kit, Thermo Fisher Scientific, USA using Quantus fluorometer, Promega, USA). The software from http://scienceprimer.com/copy-number-calculator-for-realtime-pcr was employed to count DNA copies.

### 2.6. Agarose Gel Electrophoresis

The real-time PCR products were subjected to electrophoretic analysis in 2% agarose gel in TAE buffer (20 mM sodium acetate, 1 mM EDTA, and 40 mM TRIS, pH adjusted to 7.2) using SimplySafe (Eurx, Poland). Electrophoresis was carried out at 80 V for 30 min.

## 3. Results

### 3.1. Phylogenetic Analysis and the Representative group of Betabaculovirus

**Figure 1:**
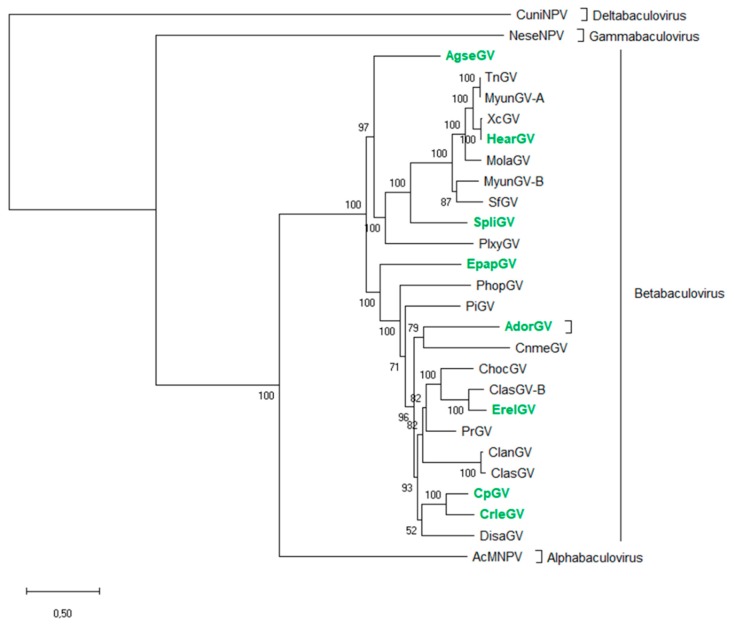
Maximum likelihood (ML) molecular phylogenetic tree based on amino acid sequences of 38 core genes of 24 baculovirus species from the *Betabaculovirus* genus and one of each of *Alpha*-, *Delta*-, and *Gammabaculovirus*. Species marked in green indicate betabaculoviruses chosen as a representative group in this study.

The phylogenetic tree based on 38 amino acid sequences of baculovirus core genes represents the general phylogeny of the *Baculoviridae* family (Figure 1), with special emphasis on the *Betabaculovirus* genus. All species included in the analysis are listed in Table 1 with the addition of host family and genome length. There are 24 betabaculovirus genomes deposited in the GenBank database, all of which differ in the nucleotide sequence (Geneious Pro 7.1 (Biomatters, Auckland, New Zealand)-MAFFT alignment. Two species, *Trichoplusia ni* granulovirus (175,360 bp, 172 orfs) and *Mythimna* (previous *Pseudaletia) unipuncta* granulovirus-A (176,677 bp, 183 orfs), share 96% of genome similarity and have almost all core genes sequences being identical (only five of them, i.e., *lef-2* (98%), *DNA polymerase* (99%), *p49* (99%), *p18* (98%), and *pif-3* (99%) share lower than 100% homology). Furthermore, these viruses cluster together on the phylogenetic tree.

Based on this molecular phylogenetic tree and the alignments of short nucleotide sequences generated from *granulin*, *lef-8*, and *lef-9* of GVs, the representative group previously described by us [32] was selected again. The selected species (Figure 1, labelled in green) differ in genomic nucleotide sequences and belong to different clades in the phylogenetic tree. Two closely related and similar betabaculoviruses, CpGV and CrleGV, were also included in the study to prove that the presented method is also able to discriminate between similar genetic sequences. The eight species (all recognized by the International Committee on Taxonomy of Viruses, ICTV) and with criteria as defined in Section 2.2, which were chosen as the demonstrative group are *Adoxophyes orana* granulovirus (AdorGV), *Agrotis segetum* granulovirus (AgseGV), CpGV, CrleGV, EpapGV, *Erinnyis ello* granulovirus (ErelGV), HearGV, and *Spodoptera litura* granulovirus (SpliGV) (bold font in Table 1 and labelled in green in Figure 1).

### 3.2. Granulin, Late Expression Factor-9, and Late Expression Factor-8 Nucleotide and Amino Acid Sequence Alignment

**Figure 2:**
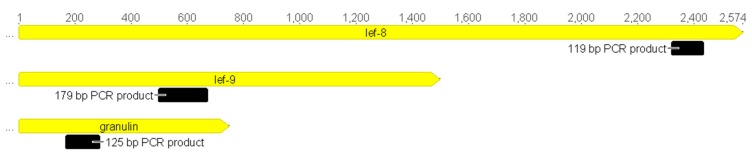
The localization of short fragments amplified in the presented real time PCR method within the *granulin*, *lef-9*, and *lef-8* genes, using AdorGV as an example.

**Figure 3:**
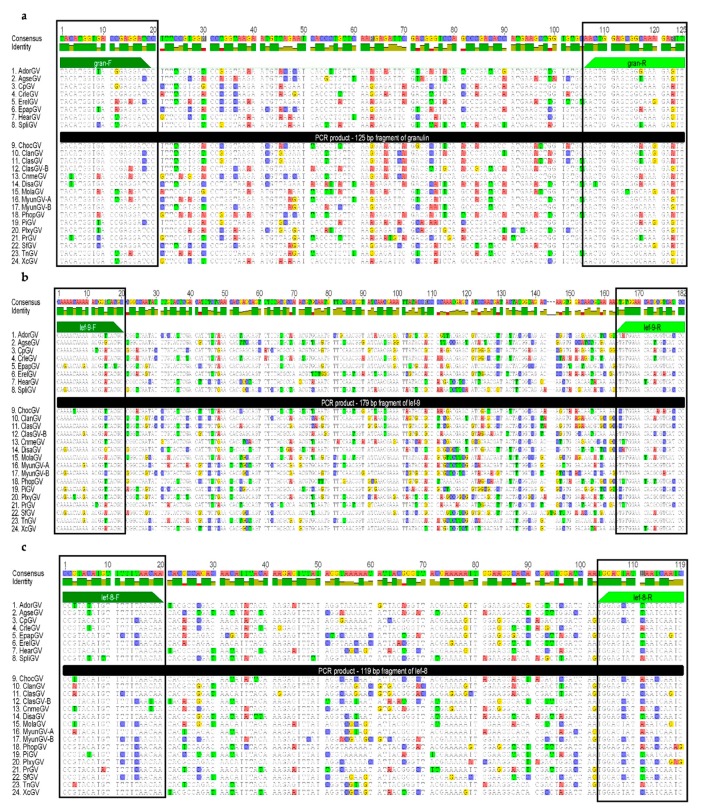
The alignment of nucleotide sequences of (**a**) *granulin* (125 bp), (**b**) *lef-9* (179 bp), and (**c**) *lef-8* (119 bp) fragments from betabaculovirus complete genomic sequences available in GenBank. 1–8 are representative groups in this study, and 9–24 are all the remaining granuloviruses. Dark and light green annotations show primers: (**a**) forward gran-F and reverse gran-R, (**b**) forward lef-9-F and reverse lef-9-R; and (**c**) forward lef-8-F and reverse lef-8-R. The histogram on the top shows the identity in every column of the alignment.

The complete nucleotide sequence of *granulin* is 747 bp long in all GVs (except for SpliGV, 750 bp) and the identity is between 70–100%. The *lef-9* gene is more variable in length, ranging from 1476 to 1512 bp with one exception: SfGV *lef-9*, which is the longest at 1632 bp. The sequence identity ranges from 54–100%. The *lef-8* codes for the biggest protein among the analyzed genes. The shortest nucleotide sequence belongs to PhopGV (2484 bp), while ClasGV-B possesses the longest *lef-8* gene of 2655 bp and the sequence identity is between 56–100%. The 100% identity between sequences is observed for TnGV and MyunGV-A. All 24 sequences (from all betabaculovirus species with complete genomic sequences in the GenBank database) were aligned and analyzed for highly conserved DNA regions that could be used as targets for degenerate primers. Highly similar sequences flank the most variable region (the localization and the size of fragments are presented in Figure 2 and fragments of the alignments are presented in Figure 3).

To show that the *gran, lef-9*, and *lef-8* regions chosen are conserved among sequenced betabaculoviruses and suitable for real-time PCR, amino acid (aa) alignments of the predicted products were performed (Figure S1). Every forward primer is coherent with codons in the *orf* and starts from the first nucleotide in the codon. Figure S1 shows high conservation of aa sequences in the annealing primer regions. For both *granulin* primers, lef-9-F and lef-8-R, the alignments are 100% similar in the aa sequence. For lef-8-F, only one aa change has occurred—the third aa, which was methionine (M) in most species and was exchanged for isoleucine (I) in two baculoviruses, SpliGV (included in the representative group) and PrGV (Figure S1b). In lef-9-R in the representative group, there are similar aa sequences in all eight tested species, but in 6 out of the remaining 16, a difference of an alanine (A) instead of valine (V) was determined (Figure S1c).

The selected, highly conserved regions were used to design a set of universal primers to amplify short fragments that are suitable in size for real-time PCR and will allow for detection of the majority of granuloviruses in less than two hours post DNA isolation. The two pairs of primers developed in the previous study [32] remain unchanged and they are universal for the larger group of granuloviruses (before—18 species, now—24 species). Therefore, they can be used in real time-PCR (Figure 3a,b). The *lef-8* alignment (Figure 3c) called for the design of a third completely new pair of degenerate primers. Other primer pairs for each gene were checked, and the results presented here represent the best (lowest degeneration and the highest specificity).

### 3.3. Real-Time PCR Assay

**Figure 4:**
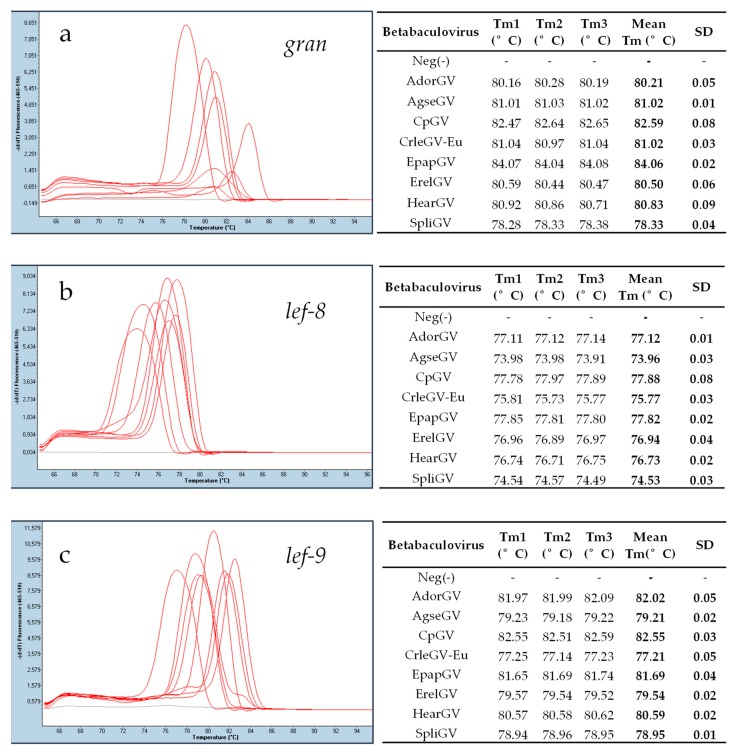
Real-time PCR results (melting curves) using universal pairs of primers that allow for the amplification of short fragments of three betabaculovirus genes: (**a**) *granulin*, (**b**) *late expression factor-8*, and (**c**) *late expression factor-9*. On the left side of (**a**), (**b**), and (**c**), melting curve analyses with red peaks from a single run (Tm1) are shown; the grey line represents a negative control [Neg(-)] with alphabaculovirus *Lymantria dispar* nucleopolyhedrovirus (LdMNPV) DNA; the *X*-axis represents temperatures; and the *Y*-axis represents –(d/dT) fluorescence (465–510 nm). On the right side of (**a**), (**b**), and (**c**), the melting temperatures (Tm) for each PCR product from three repetitions and mean Tm with SD calculated are shown.

The DNA templates from eight betabaculoviruses, AdorGV, AgseGV, CpGV, CrleGV, EpapGV, ErelGV, HearGV, and SpliGV and the three pairs of universal primers, gran-F and gran-R, lef-9-F and lef-9-R, lef-8-F and lef-8-R were used for real-time PCR reactions. The results are presented in Figure 4. Sharp peaks in melting curves correspond to specific products for all three tested genes: *granulin*, *lef-9*, and *lef-8*. Each product for a single pair of primers exhibited different melting temperatures Tm in a single run (Figure 4), which indicates that real-time PCR products have different nucleotide sequences, and the method can be used for differentiation of granuloviruses. When analyzing mean Tm (Table S1), there is one exception: The *granulin* products for AgseGV and CrleGV-Eu give the same Tm (Figure 4a). All SD values range from 0.01 to 0.09.

**Figure 5:**
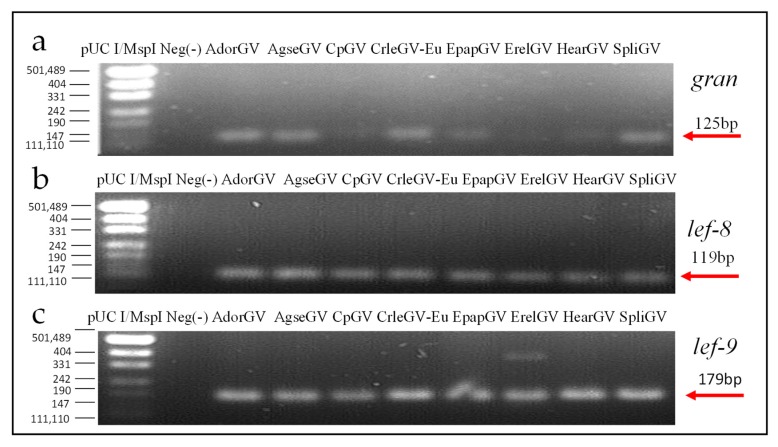
Agarose gel electrophoresis (2%) of real-time PCR products of short fragments of (**a**) *gran*—125 bp long, (**b**) *lef-8*—119 bp long, and (**c**) *lef-9*—179 bp long, after real-time PCR reactions are presented. Lane 1: pUC I/MspI DNA Ladder (Thermo Fisher Scientific, Waltham, MA, USA); lane 2: negative control with alphabaculovirus *Lymantria dispar* nucleopolyhedrovirus (LdMNPV) DNA; lanes 3–10: granuloviruses from this study.

After real-time PCR, the samples were loaded on an agarose gel (2%) to confirm the presence of specific products. All the bands were of expected sizes: 125 bp for gran, 179 bp for *lef-9*, and 119 bp for *lef-8* (Figure 5). The negative controls (with DNA from alphabaculovirus LdMNPV) are presented with grey curves in Figure 4 and in the second well of each panel on agarose gel in Figure 5.

To estimate the level of detection, the DNA of one betabaculovirus, HearGV, of known concentration was serially diluted 10^−1^–10^−10^, and the real-time PCR assay was repeated with the set of tested universal primers for granuloviruses. DNA concentration was assessed fluorometrically. For *gran*, the level of detection was 10^4^ copies of DNA molecules per reaction, and for *lef-9* and *lef-8*, it was 10^3^ copies of DNA molecules per reaction.

The real-time PCR analysis was also performed for two closely related CrleGV isolates that are commercially produced in Europe (CrleGV-Eu) and South Africa (CrleGV-SA) as components of plant protection agents (Table 2) (both originated from South Africa).

For both CrleGV isolates tested, the European and the South African, the melting temperatures differed for all three of the primer pairs used (Table 2). The largest difference in Tm was observed between the *gran* and *lef*-8 amplicons at 0.95 and 1.09 °C. The difference in Tm between the *lef-9* amplicons was not as large; however, it was well within the sensitivity of the real-time PCR thermocycler used. Polymorphism sites that exist in sequences may or may not introduce a detectable change in Tm, which will be a cumulative effect of nucleotide type and position in the whole sequence. Therefore, as expected, no correlation between the number of single nucleotide polymorphism sites (SNPs) and the |Δ Mean Tm| was observed. When multiple SNPs exist, they can increase (here for *lef-8*) or decrease (here for *lef-9*) ΔTm in the compared sequences.

## 4. Discussion

**Figure 6:**
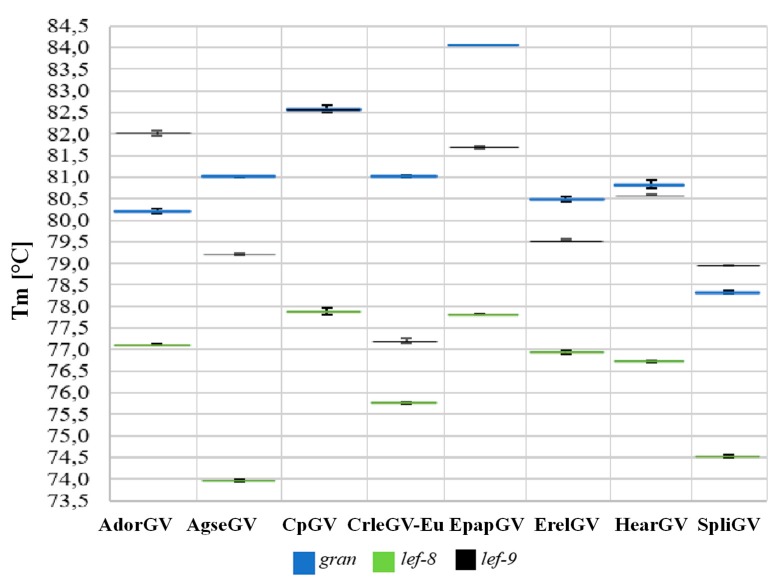
Schematic representation of the results of the described real-time PCR-based method for differentiation of betabaculovirus species based on three genes: *gran, lef-8, and lef-9*. Each line represents Tm for real-time PCR products (*gran*—blue, *lef-8*—green, and *lef-9*—black) with SD values.

The primary objective of the presented study was to develop a fast and easily accessible method for granulovirus detection and differentiation. For this purpose, a real-time PCR technique was chosen. Real-time PCR is commonly used for the detection of many different viruses, from various families, that infect humans, animals, or bacteria [26,35,36]. In the case of insect pathogens, the method based on real-time PCR was developed for sensitive detection and quantitative analysis of larvae infected with one granulovirus: EpapGV [37]. The method proposed in this study allows for the discrimination of different GVs and gives comparable results with the one based on the MSSCP technique described by us previously [33] (Figure 6).

The comparison of Tm from three amplified fragments between each other for all tested species has been done and presented in Table S1 as absolute values from the Tm difference between each species (e.g., AdorGV Tm_gran_ compared with AgseGV Tm_gran_). For granulovirus species from this study, the Tm in a single run for each of three genes differs. In addition, when the Tm values for each gene in one species are compared with the Tm values for each gene in another species, there is always at least one high Tm difference (a ≥ 0.3 °C difference between Tm was assigned as “high”, and “low” values (<0.3 °C) are marked in red in Table S1). It is therefore possible, when differentiation is not based on one gene alone but on all three genes, to differentiate between the granulovirus species tested using the method described. Except for AdorGV and EpapGV (*gran* and *lef-8*), all the other absolute value Tm differences are higher than 0.3 °C for at least two out of three genes. For two species, AgseGV and CrleGV, while there was no difference between the mean Tm for *gran* fragments, the absolute values of Tm difference for the remaining two genes are well above the value of sensitivity of detection, which is 0.01 °C, with *lef-8* at 1.81 °C and *lef-9* at 2 °C. Hence, the proposed procedure may be very useful as a screening method, as the high differentiation levels provide a greater margin, which allows for easy application of this method in numerous real-time thermocyclers from different vendors.

*Granulin/polyhedrin*, *late expression factor-8*, and *late expression factor-9* belong to the most conserved group of genes among baculoviruses. *Lef-8* and *lef-9* are core genes present in every species, while *granulin*, though not coded by a core gene, has highly conserved structure and resembles another major occlusion body protein—polyhedrin (30 kDa)—found in nucleopolyhedroviruses. Only the *Deltabaculovirus* group, which, according to phylogeny, is older and more distant from lepidopteran-specific baculoviruses (therefore closer to a common ancestor) possesses a gene coding for a trimer built of polypeptides of 30 kDa which is non-homologous towards the *polyhedrin* or *granulin* genes [22,38,39]. The lepidopteran-specific baculovirus demarcation criterion that is commonly used for species designation includes Kimura-two parameter (K2P) nucleotide distance comparisons based on partial sequences of *polh/gran*, *lef-8*, and *lef-9* [25]. Recently, it has been extended for larger group of genes, i.e., all core genes, and the grouping of baculoviruses has not changed based on the larger number of genes. In general, the criterion mentioned was confirmed by the distance of 38 common gene nucleotide sequences [40], which means that species defined based on fragments of these three genes remain unchanged for known members of the *Baculoviridae* family. Therefore, *gran, lef-8*, and *lef-9* genes selected for this study are an ideal choice for betabaculovirus determination.

The number of completely sequenced granulovirus species (24) is still low in comparison to nucleopolyhedroviruses (73) (GenBank, August 2018). The possibility that the primers will not match sequences of some of the newly discovered isolates/species cannot be excluded. The nucleotide and amino acid (Figure S1) sequences of available species were analyzed. Although the representative group consists of eight members of *Betabaculovirus*, which equals one-third part of all completely sequenced species, we hypothesize that it represents the majority of known GVs, with the main limitation encountered being DNA availability. However, the regions selected for degenerate primer targets have highly conserved protein structure, so in future, the method may find application for the detection and characterization of new granuloviruses for samples originating from the environment.

It is common knowledge that many pathogens of humans and animals, including viruses infecting insects, possess high levels of genotypic variation [41,42]. Many variants of baculoviruses circulate in one host or can be isolated from the same species from distinct geographic localizations. The genotype sequences may be characterized with single nucleotide polymorphisms (SNPs), substitutions, and indels (insertion/deletion) that do or do not cause *orf* changes [43,44,45,46,47]. Baculovirus variability in genomes should be carefully investigated because of selection pressure due to biopesticide application in the field. Using standard PCR with bulk products and sequencing may not be necessary to detect minor variants that may affect virus stability and biopesticide efficacy. Over a decade ago, after around 20 years of field application of commercial insecticide based on CpGV, resistance development in caterpillars has appeared in orchards in France and Germany [48]. To date, three mechanisms of resistance have been described [48,49,50,51,52,53]. Taking such a possibility into account for other viral biopesticides, the monitoring of isolates and variants occurring in caterpillars should be implemented routinely in biopesticide production. The proposed real-time PCR-based method shows that it is possible to distinguish two CrleGV isolates originated from South Africa on the basis of three genes. The method could likely be used to detect minor variants in the virus population (even another closely related granulovirus species).

We have previously developed a method based on the Multi-temperature Single Stranded Conformational Polymorphism (MSSCP) technique, which has advantages similar to the one described in this study [33]. However, the MSSCP-based method has some drawbacks: It requires dedicated equipment, dealing with polyacrylamide gel, and DNA silver staining. In the current work, we developed a method for discriminating between the representative groups of betabaculoviruses using a real-time thermocycler, which may be more accessible to many laboratories in contrast to the MSSCP technique. Real-time thermocyclers are increasingly more affordable (e.g., a single channel costs ~4299 USD) and are starting to become standardized equipment in many laboratories, with methods that make use of them therefore becoming considerably more implementable. Obviously, NGS-based methods supply a wealth of data, allowing for the detection and differentiation of baculoviruses, but the costs of sequencing runs and the amount of data needed to be processed is still too high for routine monitoring of infected insects. Furthermore, this method is particularly advantageous when screening many samples, allowing detection and differentiation without the need for amplicon purification, sequencing, and bioinformatic analysis. Therefore, the method presented in this study may find extensive application in laboratories screening large batch samples of current or new betabaculoviruses, as we estimate a cost per sample around 2 USD.

## Figures and Tables

**Table 1:** List of betabaculoviruses (24 species) which have complete genomic sequences available in GenBank (August 2018). Granuloviruses (GVs) chosen as the representative group in this study are shown in bold.

Abrreviation	Betabaculovirus Name	Host Family	GenBank Accession Number	Genome Size (bp)
**AdorGV**	*Adoxophyes orana* granulovirus	Tortricidae	AF547984	99,657
**AgseGV**	*Agrotis segetum* granulovirus	Noctuidae	AY522332	131,680
ChocGV	*Choristoneura occidentalis* granulovirus	Tortricidae	DQ333351	104,710
ClanGV	*Clostera anachoreta* granulovirus	Notodontidae	HQ116624	101,487
ClasGV	*Clostera anastomosis* granulovirus	Notodontidae	KC179784	101,818
ClasGV-B	*Clostera anastomosis* granulovirus B	Notodontidae	KR091910	107,439
CnmeGV	*Cnaphalocrocis medinalis* granulovirus	Crambidae	KU593505	111,246
**CpGV**	*Cydia pomonella* granulovirus	Tortricidae	U53466	123,500
**CrleGV**	*Cryptophlebia leucotreta* granulovirus	Tortricidae	AY229987	110,907
DisaGV	*Diatraea saccharalis* granulovirus	Crambidae	KP296186	98,392
**EpapGV**	*Epinotia aporema* granulovirus	Tortricidae	JN408834	119,082
**ErelGV**	*Erinnyis ello* granulovirus	Sphingidae	KJ406702	102,759
**HearGV**	*Helicoverpa armigera* granulovirus	Noctuidae	EU255577	169,794
MolaGV	*Mocis latipes* granulovírus	Noctuidae	KR011718	134,272
MyunGV-A	*Mythimna unipuncta* granulovirus A	Noctuidae	EU678671	176,677
MyunGV-B	*Mythimna unipuncta* granulovírus B	Noctuidae	KX855660	144,673
PhopGV	*Phthorimaea operculella* granulovirus	Gelechiidae	AF499596	119,217
PiGV	*Plodia interpunctella* granulovirus	Pyralidae	KX151395	112,536
PlxyGV	*Plutella xylostella* granulovirus	Plutellidae	AF270937	100,999
PrGV	*Pieris rapae* granulovirus isolate	Pieridae	GQ884143	108,592
SfGV	*Spodoptera frugiperda* granulovirus	Noctuidae	KM371112	140,913
**SpliGV**	*Spodoptera litura* granulovirus	Noctuidae	DQ288858	124,121
TnGV	*Trichoplusia ni* granulovirus	Noctuidae	KU752557	175,360
XcGV	*Xestia c-nigrum* granulovirus	Noctuidae	AF162221	178,733

**Table 2:** Melting temperatures from real-time PCR analysis using universal pairs of primers that allow for amplification of short fragments of *gran*, *lef-8*, and *lef-9* on the DNA of CrleGV isolates—European (Eu) and South African (SA). Tm from three repetitions with mean Tm and SD are shown. The negative control (Neg(-)) included alphabaculovirus LdMNPV DNA. The absolute values from the Tm difference between both isolates are shown. SNPs—single nucleotide polymorphism sites.

	Isolate	Tm1 (°C)	Tm2 (°C)	Tm3 (°C)	Mean Tm (°C)	SD	|ΔMean Tm| (°C)	SNPs
*gran*	Neg(-)	-	-	-	-	-	-	
	CrleGV-Eu	80.97	81.04	81.04	81.02	0.03	0.95	1
	CrleGV-SA	82.05	81.92	81.95	81.97	0.06		
*lef-8*	Neg(-)	-	-	-	-	-	-	
	CrleGV-Eu	75.73	75.77	75.81	75.77	0.03	1.09	2
	CrleGV-SA	76.89	76.85	76.84	76.86	0.02		
*lef-9*	Neg(-)	-	-	-	-	-	-	
	CrleGV-Eu	77.14	77.23	77.25	77.21	0.05	0.29	3
	CrleGV-SA	77.53	77.45	77.53	77.50	0.04		

## Supplementary Materials

The following are available online at http://www.mdpi.com/1999-4915/11/2/115/s1. Table S1: The absolute values from the Tm difference between each species (e.g., AdorGV Tm *gran* compared with AgseGV Tm *gran*). Figure S1: The amino acid sequence alignments of predicted PCR products with primers: (a) *gran*, (b) *lef-8*, and (c) *lef-9*. 1–8 are the representative group in this study, 9–24 are all the remaining granuloviruses.
