# Supplementary material for: New Method for Differentiation of Granuloviruses (Betabaculoviruses) Based on Real-Time Polymerase Chain Reaction (Real-Time PCR)
Source: Viruses. 2019 Jan 29;11(2):115. doi: 10.3390/v11020115 (PMC6410086; doi:10.3390/v11020115)
Supplement: Supplementary file 1 [file viruses-11-00115-s001.zip › Appendix Viruses.docx]

**Table A1.** The absolute values from the Tm difference between each species (e.g. AdorGV Tm *gran* compared with AgseGV Tm *gran*). The differences below 0.3 °C were labelled in red. The species chosen for comparison of the temperatures are marked in green.

|  | ***gran*** (\|ΔTm\| [̊C]) | ***lef-8*** (\|ΔTm\| [̊C]) | ***lef-9*** (\|ΔTm\| [̊C]) |
| --- | --- | --- | --- |
| **AdorGV** | 0 | 0 | 0 |
| AgseGV | 0.81 | 3.16 | 2.81 |
| CpGV | 2.38 | 0.76 | 0.53 |
| CrleGV-Eu | 0.81 | 1.35 | 4.81 |
| ErelGV | 3.85 | 0.7 | 0.33 |
| EpapGV | 0.29 | 0.18 | 2.48 |
| HearGV | 0.62 | 0.39 | 1.43 |
| SpliGV | 1.88 | 2.59 | 3.07 |
|  |  |  |  |
|  | ***gran*** (\|ΔTm\| [̊C]) | ***lef-8*** (\|ΔTm\| [̊C]) | ***lef-9*** (\|ΔTm\| [̊C]) |
| AdorGV | 0.81 | 3.16 | 2.81 |
| **AgseGV** | 0 | 0 | 0 |
| CpGV | 1.57 | 3.92 | 3.34 |
| CrleGV-Eu | 0 | 1.81 | 2 |
| ErelGV | 3.04 | 3.86 | 2.48 |
| EpapGV | 0.52 | 2.98 | 0.33 |
| HearGV | 0.19 | 2.77 | 1.38 |
| SpliGV | 2.69 | 0.57 | 0.26 |
|  |  |  |  |
|  | ***gran*** (\|ΔTm\| [̊C]) | ***lef-8*** (\|ΔTm\| [̊C]) | ***lef-9*** (\|ΔTm\| [̊C]) |
| AdorGV | 2.38 | 0.76 | 0.53 |
| AgseGV | 1.57 | 3.92 | 3.34 |
| **CpGV** | 0 | 0 | 0 |
| CrleGV-Eu | 1.57 | 2.11 | 5.34 |
| ErelGV | 1.47 | 0.06 | 0.86 |
| EpapGV | 2.09 | 0.94 | 3.01 |
| HearGV | 1.76 | 1.15 | 1.96 |
| SpliGV | 4.26 | 3.35 | 3.6 |
|  |  |  |  |
|  | ***gran*** (\|ΔTm\| [̊C]) | ***lef-8*** (\|ΔTm\| [̊C]) | ***lef-9*** (\|ΔTm\| [̊C]) |
| AdorGV | 0.81 | 1.35 | 4.81 |
| AgseGV | 0 | 1.81 | 2 |
| CpGV | 1.57 | 2.11 | 5.34 |
| **CrleGV-Eu** | 0 | 0 | 0 |
| ErelGV | 3.04 | 2.05 | 4.48 |
| EpapGV | 0.52 | 1.17 | 2.33 |
| HearGV | 0.19 | 0.96 | 3.38 |
| SpliGV | 2.69 | 1.24 | 1.74 |
|  |  |  |  |
|  | ***gran*** (\|ΔTm\| [̊C]) | ***lef-8*** (\|ΔTm\| [̊C]) | ***lef-9*** (\|ΔTm\| [̊C]) |
| AdorGV | 3.85 | 0.7 | 0.33 |
| AgseGV | 3.04 | 3.86 | 2.48 |
| CpGV | 1.47 | 0.06 | 0.86 |
| CrleGV-Eu | 3.04 | 2.05 | 4.48 |
| **ErelGV** | 0 | 0 | 0 |
| EpapGV | 3.56 | 0.88 | 2.15 |
| HearGV | 3.23 | 1.09 | 1.1 |
| SpliGV | 5.73 | 3.29 | 2.74 |
|  |  |  |  |
|  | ***gran*** (\|ΔTm\| [̊C]) | ***lef-8*** (\|ΔTm\| [̊C]) | ***lef-9*** (\|ΔTm\| [̊C]) |
| AdorGV | 0.29 | 0.18 | 2.48 |
| AgseGV | 0.52 | 2.98 | 0.33 |
| CpGV | 2.09 | 0.94 | 3.01 |
| CrleGV-Eu | 0.52 | 1.17 | 2.33 |
| ErelGV | 3.56 | 0.88 | 2.15 |
| **EpapGV** | 0 | 0 | 0 |
| HearGV | 0.33 | 0.21 | 1.05 |
| SpliGV | 2.17 | 2.41 | 0.59 |
|  |  |  |  |
|  | ***gran*** (\|ΔTm\| [̊C]) | ***lef-8*** (\|ΔTm\| [̊C]) | ***lef-9*** (\|ΔTm\| [̊C]) |
| AdorGV | 0.62 | 0.39 | 1.43 |
| AgseGV | 0.19 | 2.77 | 1.38 |
| CpGV | 1.76 | 1.15 | 1.96 |
| CrleGV-Eu | 0.19 | 0.96 | 3.38 |
| ErelGV | 3.23 | 1.09 | 1.1 |
| EpapGV | 0.33 | 0.21 | 1.05 |
| **HearGV** | 0 | 0 | 0 |
| SpliGV | 2.5 | 2.2 | 1.64 |
|  |  |  |  |
|  | ***gran*** (\|ΔTm\| [̊C]) | ***lef-8*** (\|ΔTm\| [̊C]) | ***lef-9*** (\|ΔTm\| [̊C]) |
| AdorGV | 1.88 | 2.59 | 3.07 |
| AgseGV | 2.69 | 0.57 | 0.26 |
| CpGV | 4.26 | 3.35 | 3.6 |
| CrleGV-Eu | 2.69 | 1.24 | 1.74 |
| ErelGV | 5.73 | 3.29 | 2.74 |
| EpapGV | 2.17 | 2.41 | 0.59 |
| HearGV | 2.5 | 2.2 | 1.64 |
| SpliGV | 0 | 0 | 0 |

**c**

**b**

**a**

**Figure A1**. The amino acid sequence alignments of predicted PCR products with primers: (a) *gran* (b) *lef-8* (c) *lef-9*, 1-8 are the representative group in this study, 9-24 are all the remaining granuloviruses. Dark and light green annotations show primers: (a) forward gran-F and reverse gran-, (b) forward lef-8-F and reverse lef-8-R; (c) forward lef-9-F and reverse lef-9-R, respectively. The histogram on the top shows the identity in every column of the alignment. The additional aa before the beginning of the reverse primers is because the length of the products is not a multiple of 3; aa mismatches are marked with a black circle.
